# MALDI imaging combined with two-photon microscopy reveals local differences in the heterogeneity of colorectal cancer

**DOI:** 10.1038/s44303-024-00041-3

**Published:** 2024-09-23

**Authors:** Arora Bharti, Kulkarni Ajinkya, Markus M. Andrea, Ramos-Gomes Fernanda, Bohnenberger Hanibal, Ströbel Philipp, Alves Frauke, Klein Oliver

**Affiliations:** 1 Translational Molecular Imaging, Max-Planck-Institute for Multidisciplinary Sciences, Hermann Rein ‑Straße 3, 37075 Göttingen, Germany; 2 Institute of Pathology, University Medical Center Göttingen, Robert-Koch-Straβe 40, 37075 Göttingen, Germany; 3 Clinic for Haematology and Medical Oncology, Institute of Interventional and Diagnostic Radiology, University Medical Center Göttingen, Robert-Koch-Straβe 40, 37075 Göttingen, Germany; 4 Cluster of Excellence “Multiscale Bioimaging: from Molecular Machines to Networks of Excitable Cells” (MBExC), University of Goettingen, Goettingen, Germany; 5 Berlin Institute of Health at Charité – Universitaetsmedizin Berlin, Core Unit Imaging Mass Spectrometry, 13353 Berlin, Germany

**Keywords:** Cancer, Image processing, Imaging, Microscopy

## Abstract

Colorectal cancer (CRC) remains a leading cause of cancer-related mortality worldwide, accentuated by its heterogeneity and complex tumour microenvironment (TME). The role of TME on tumour pathophysiology is pivotal, especially the influence of components of the extracellular matrix (ECM), such as collagen. We introduce a novel multimodal imaging strategy to unravel the complex spatial heterogeneity of CRC by integrating the imaging features from two-photon laser scanning microscopy (2PLSM) and histology with proteomics signatures from matrix-assisted laser desorption ionization-mass spectrometry imaging (MALDI MSI). Our study is the first to correlate the structural coherence of collagen fibres and the nuclei distribution profile of tumour tissue with the peptide signatures, offering insights into the proteomic landscape of CRC within regions of high nuclei distribution (HND), as well as chaotic and organised regions of collagen. We use this approach to distinguish the patient tissues originating from left-sided colorectal cancer (LSCC) and from right-sided colorectal cancer (RSCC). This discriminative signature highlights the role of high nuclei distribution and collagen architecture in tumour progression. Complementary *m/z* values of several proteins associated to components of ECM, such as plectin, vinculin, vimentin, and myosin, have shown differentially intensity distributions between LSCC and RSCC. Our findings demonstrate the potential of combining structural information with peptide features to identify molecular signatures in different tumour regions and retrieve new insights into CRC pathophysiology.

## Introduction

The GLOBOSCAN 2020 report by the World Health Organization calls attention to the increasing number of colorectal cancer (CRC) cases in the world, with the third-highest incidence rate and the second-highest mortality rate in the world^1^. These cases have been predicted to increase by 71.5% by 2035^2^. Even though the role of the tumour microenvironment (TME) in influencing cancer progression, metastasis, and response to chemoradiotherapy is increasingly recognised, the spatial heterogeneity of the TME still poses a problem for both subtyping and therapy^3,4^. The influence of the extracellular matrix (ECM), particularly collagen, on the tumour pathophysiology is also apparent^5,6^. Histology with haematoxylin and eosin (H&E) staining, along with colonoscopy, x-ray imaging, and molecular screening (mutations in KRAS or microsatellite instability), are routinely used methods for monitoring the prognosis of CRC in clinics^7,8^. However, these methods cannot provide the characterisation of the spatial complexity of tumour tissues, such as the interplay of cancer proteome, collagen architecture, and alterations in the nuclei distribution that influence the tumour pathophysiology.

Two-photon laser scanning microscopy (2PLSM) allows a label-free imaging approach that captures the intrinsic second harmonic generation (SHG) signal emitted by non-centrosymmetric molecular structures, notably collagen fibres^9^. The two-photon excitation autofluorescence (2PEF) signal concurrently emitted by the tissue gives information on the tissue morphology. Therefore, 2PLSM enables the visualisation of collagen fibre architecture within the tissues at sub-micron resolution without necessitating extrinsic fluorophores or a staining process^10^. Recent investigations have underscored the significance of collagen fibre-associated imaging features in various clinical aspects of CRC, such as predicting a pathological complete response^11^, providing the immunoscore^12^, an association of collagen signatures with lymph node metastasis^13^ and determining the prognosis of CRC patients. A recent study has shown differences in the collagen morphology in the ECM of CRC based on the anatomical location of the tumour, distinguishing between LSCC and RSCC by the higher collagen fibre content found in LSCC^14^.

Despite these advancements, a comprehensive understanding of how collagen features influence tumour pathophysiology remains elusive. Specifically, there is a notable gap in research regarding the spatial correlation between collagen-associated imaging features and the protein landscape in ECM structures. Thus, to further understand the mechanism by which specific collagen features affect tumour pathophysiology, identifying the proteins involved in the dynamic remodelling of ECM during tumour progression is of prime importance.

MALDI mass spectrometry imaging (MALDI MSI) combines spatial and label-free molecular profiling and histological evaluation directly in tissue sections^15,16^. This imaging modality combines the capability of mass spectrometry to identify molecules (peptides, lipids, drugs, and other compounds) while preserving spatial information^17^. Recent studies have exploited MALDI MSI in deciphering CRC pathophysiology, including i) the identification of proteomics signatures that can help to predict distant metastasis in CRC patient tissues^18^, ii) the correlation of proteomics signatures to clinical outcome in CRC patient tissue microarrays^19^, iii) the combination with chemometric analysis to discriminate CRC tumour cell populations^20^, and iv) even the report of abnormal phospholipids in CRC liver metastasis^21^.

However, narrowing down the relevant peptides of interest from an extensive array of detected proteomics signatures poses a challenge. In this study, we present a new approach to characterise distinctive areas of the TME of CRC patient tissues by combining, for the first time, imaging features derived from 2PLSM and histology with proteomics signatures from MALDI MSI.

We first derived a heatmap of the coherence in the collagen fibre organisation in tumour tissues before performing MALDI MSI, followed by generating heatmaps of the nuclei distribution in the tissue by using histology. These imaging biomarkers were used to refine the proteomic signatures detected by the MALDI MSI. We present this approach of correlative MALDI MSI and 2PLSM exemplary for the examination of differences between the proteomics signatures of the left-sided colorectal cancer (LSCC) and the right-sided colorectal cancer (RSCC).

## Material and methods

### Tumour sample collection

The study cohort consists of human tumour tissues resected from patients with CRC (*n* = 14). The cohort has a balanced distribution of the anatomical origin of the tumour, stages and morphology of the tumour, as detailed in the sample information table (Table 1: Sample information). The samples were collected and used according to the approval and regulations of the Ethics Committee of the University Medical Center Goettingen (Ethics approval: #5/10/17). All patients provided written informed consent for using pathology specimens for research purposes. The study was performed in accordance with the Declaration of Helsinki.

**Table 1:** Sample information

S.No.	TNM classification	Gender	Age	TNM stage	Grade (G2/G3)	LSCC/RSCC
1	pT3 N0 (0/19) L0 V0 Pn0 G2 R0	M	83	T3	G2	RSCC
2	pT3 N1a (1/38) L1 V0 Pn0 G2 R0	F	88	T3	G2	RSCC
3	pT2 N0 (0/12) L1 V0 Pn0 G3 R0	M	81	T2	G3	RSCC
4	pT1 N0 (0/28) G2 R0	F	73	T1	G2	RSCC
5	pT3 N0 (0/25) M1 (PER) L1 V0 Pn0 G3 R0	M	49	T3	G3	RSCC
6	pT2 N1b (3/88) L0 V0 G2 R0	F	49	T2	G2	RSCC
7	pT3 N2a (6/47) L1 V0 G3 R0	F	75	T3	G3	LSCC
8	pT3 N0 (0/15) G2 R0	M	72	T3	G2	LSCC
9	pT3 N0 (0/15) L0 V1 Pn1 G2 R0	M	66	T3	G2	LSCC
10	pT2 N0 (0/12) L0 V0 Pn0 G2 R0	M	73	T2	G2	LSCC
11	pT3 N2b (8/25) M1a (HEP) L1 V1 Pn1 R0 G2	M	81	T3	G2	LSCC
12	pT3 N0 (0/16) L0 V0 G2 R0	M	59	T3	G2	LSCC
13	pT2 N0 (0/19) MX L0 V0 Pn0 G2 R0	F	66	T2	G2	LSCC
14	pT3 N1a (1/17) L0 V0 Pn0 R0 G2	M	95	T3	G2	LSCC

The tumours have been categorised by the TNM staging, where T denotes the stage (from T1 to T4 in the ascending order of aggressiveness), N indicates lymph node involvement, and M indicates the metastasis status. The grade gives information on the appearance of tumour cells, where G1 indicates well-differentiated tissues, G2 is moderately differentiated, and G3 is poorly differentiated tissues. All the tumours are microsatellite stable (MSS).

### Label-free imaging

2PLSM images were acquired using an upright TriM Scope II multiphoton microscope (Miltenyi Biotec, Bielefeld, Germany) equipped with a tunable femtosecond laser (Cronus 2 P, Light Conversion, Vilnius, Lithuania). The laser was tuned to 870 nm at 15% of its maximal output power (1.0 W) for SHG and 2PEF excitation. Images were acquired using an Olympus XLPLAN 25x (NA 1.05) water immersion objective. The backscattered emitted light was split by 495 nm and 560 nm long pass dichroic mirrors (Semrock) and detected through the objective lens at photomultiplier tube (PMT) and GaAsP detectors (Hamamatsu). A PMT in the transmission position collected the forward scattered emitted light gathered by a 1.4 NA condenser lens under the stage. SHG and 2PEF signals were collected using the filter settings 434 ± 20 nm and 525 ± 50 nm, respectively (BrightLine HD filters, Semrock, AHF Analysentechnik Tübingen, Germany). Collagen fibre signals were collected as forward scattered SHG (F-SHG). The 2PEF signal was collected from backscattered photons. 2D mosaics of entire sections were acquired with individual image sizes of 393 × 393 µm with 1024 × 1024 pixels, a frequency of 600 Hz with 2-fold averaging, and 10% overlap within each tile of the mosaic.

### MALDI IMS sample preparation

All tissue samples were fixed in 4% paraformaldehyde and embedded in paraffin. 5 µm thick sections were cut from the paraffin blocks using a vibratome (VT1000 S; Leica Biosystems) and mounted on conductive glass slides coated in indium tin oxide (Bruker Daltonik GmbH, Bremen, Germany). The sections on the slides were covered with a coverslip before imaging by 2PLSM. After 2PLSM imaging, the coverslip was removed, and the samples were prepared for MALDI MSI as detailed by Wu et al.^24^. Sections were preheated to 80 °C for 15 min. Deparaffinisation was performed by successive immersion in xylene, 100% isopropanol, and successive hydration steps of 100%, 96%, 70%, and 50% ethanol for 5 minutes each. Heat-induced antigen retrieval was performed in MilliQ-water in a steamer for 20 min. After drying the slides for 10 min, tryptic digestion was performed using an automated spraying device (HTX TM-Sprayer, HTX Technologies LLC, ERC GmbH, Riemerling, Germany). 16 layers of tryptic solution (20 µg Promega® Sequencing Grade Modified Porcine Trypsin in 800 µL digestion buffer-20 mM ammonium bicarbonate with 0.01% glycerol, flow rate = 0.015 ml/min velocity = 750 mm/min, track spacing: 2 mm, concentration = 0.025 mg/ml, pattern CC) were sprayed onto each section at 30 °C. Tissue sections were then incubated for 2 h at 50 °C in a humidity chamber saturated with potassium sulphate solution. After incubation, the HTX TM Sprayer applied 4 layers of the matrix solution (7 g/L a-cyano-4-hydroxycinnamic acid in 70% acetonitrile and 1% trifluoroacetic acid, flow rate = 0.012 ml/min velocity = 1200 mm/min, track spacing: 3 mm, concentration = 7 mg/ml, pattern HH) at 75 °C.

### MALDI MSI

MALDI imaging was performed on the rapifleX® MALDI Tissuetyper® (Bruker Daltonik GmbH, Bremen, Germany) in reflector mode with a detection range of 800–3200 *m/z*, 500 laser shots per spot, a 1.25 GS/s sampling rate and raster width of 50 µm. FlexImaging 5.1 and flexControl 3.0 software (Bruker Daltonik GmbH) were used in coordination. External calibration was performed using a peptide calibration standard (Bruker Daltonik GmbH).

### Protein identification

Identification of proteins based on *m/z* value was conducted on adjacent tissue sections utilising a bottom-up approach involving nano-liquid chromatography electrospray ionization tandem mass-spectrometry, as outlined in previous work^22^. Firstly, the samples were preheated for 15 min at 80 °C, followed by deparaffinization, antigen retrieval, and tryptic digestion. The samples were then incubated for 2 h at 50 °C in a humidity chamber filled with a potassium sulphate solution. Peptides were separately extracted from each tissue after 2 h into 40 µL of 0.1% trifluoroacetic acid (TFA) and then incubated for 15 min at room temperature. As per the manufacturer’s guidelines, the digests were filtered through a ZipTip®C18, and the filtrates were concentrated using a vacuum evaporator (Eppendorf® Concentrator 5301, Eppendorf AG, Germany) and re-dissolved in 0.1% TFA. 2 µL of the peptide mix was loaded to an Acclaim PepMap™ 100 C18 trap column (100 µm × 2 cm, PN 164564, Thermo Fisher Scientific, USA) and pre-treated with 10 mM sodium hypofluorite (flow rate 20 µL/h) before being separated on an Acclaim PepMap™ RSLC C18 column (75 µm × 50 cm, PN 164942, Thermo Fisher Scientific, USA) using a 2–35% acetonitrile gradient in 0.1% formic acid (flow rate 400 nL/min, pressure range 10–800 bar) over 90 min at 60 °C. The tandem mass spectrometer (Impact II, ESI QTOF MS, Bruker Daltonik GmbH, Bremen, Germany) detected the ionized peptides through a full-mass scan (150–2200 *m/z*) at a 50,000 FWHM resolution. The autoMS/MS Insant Expertise feature selected peaks for fragmentation via collision-induced dissociation. For peptide identification, the peak lists were searched in the human Swiss-Prot database using PEAKS-studio-proteomics software (Bioinformatics Solutions, Version 11.6) using PEAKS-DB and PEAKS-de-novo sequencing. For MALDI MSI and LC–MS/MS *m/z* value comparisons, identification required more than one peptide with mass differences <0.15 Da. Peptides that had the highest score (-10lolgP, protein confidence score) and the smallest mass differences between the MALDI MSI and LC-MS/MS datasets were assumed as identified. Deisotoping was performed.

### Histology

Following MALDI -MSI, the matrix was removed from the tissue sections with 70% ethanol, and the sections were then washed in distilled water for 7 min. They were stained with haematoxylin (Himedia) for 10 min and washed with warm water twice for 10 min each. This was followed by washing with distilled water. The slides were then stained with eosin (Himedia) for 60 s and washed twice with distilled water. Serial dehydration was performed by immersing the slides in 80%, 96%, 100% ethanol, and xylol, followed by mounting them with a coverslip. The slides were then scanned with a whole-section scanner (NanoZoomer, Hamamatsu). These haematoxylin and eosin (H&E) images were used to annotate tumour regions in Qupath SW^23^ by a pathologist before transferring them into SCiLS Lab software version 2024a Pro (12.00.15110).

### Image processing

The F-SHG signal from 2PLSM images was extracted and denoised using the global Otsu adaptive thresholding. Texture analysis was performed on the SHG signal as explained before^14^ to quantify the coherence of collagen fibre. The local coherence heatmap is bound between values 0 and 1, where 1 indicates highly aligned structures, and 0 indicates chaotic and disorganised fibres. This coherence map was overlaid on the original 2PLSM image in FIJI by merging images to stack, followed by taking a maximum intensity projection of the merged stack. The coherence regions were classified as chaotic (local coherence values from 0 to 0.5) and organised (local coherence values from 0.5 to 1). The percentage area occupied by chaotic and organised regions was quantified.

The nuclei were segmented from the H&E images using the “2D_versatile_he”pre-trained model from StarDist^24^. During post nuclei segmentation, the nuclei distribution was quantified by identifying the number of nuclei per unit area. A heatmap of nuclei distribution was formed, bound between 0 and 1, where 1 indicates densely packed nuclei, and 0 indicates nuclei scattered largely in space. This nuclei distribution map was then overlaid on the original H&E image in FIJI by merging images to stack, followed by taking a maximum intensity projection of the merged stack. The nuclei distribution regions were classified as low nuclei distribution (values from 0 to 0.5) and high nuclei distribution (values from 0.5 to 1). The percentage area occupied by low and high nuclei distribution regions was quantified. The nuclei segmentation and texture analysis source codes are available at dedicated GitHub repositories^25,26^.

### STRING analysis

Protein-protein associations were derived from the STRING database (https://string-db.org/). The list of UniProt IDs of peptides identified by MALDI MSI was added in the ‘Multiple proteins’ search for the organism *Homo sapiens* and the confidence interval score of 0.4 to generate a full string network indicating both functional and physical edges. The thicker edge indicates the strength of the data support. Functional enrichment analysis was performed by applying the following settings: Maximum FDR < 0.01, strength >0.01 and minimum count in network.

### MALDI MSI data processing for statistics

MALDI MSI raw data were imported into the SCiLS Lab software version 2024a Pro (Bruker Daltonik GmbH) using settings to preserve the total ion count without baseline removal and converted into the SCiLS base data .sbd and .slx file. An attribute table was made, which consists of annotated regions of LSCC, RSCC, high collagen coherence, low collagen coherence, high nuclei distribution and low nuclei distribution. These attributes were used to divide the dataset into respective spatial groups to compare the differences in the spectra in particular regions of the tissues. Peak finding and alignment were conducted across a dataset using a standard segmentation pipeline (SciLS Lab software) in maximal interval processing mode with TIC normalisation, medium noise reduction and no smoothing. Annotated regions were transferred from QuPath as sef.file into SCilS Lab.

### Statistics evaluation

Discriminative MALDI MSI *m/z* values from annotated regions of each group (LSCC, RSCC, high collagen coherence, low collagen coherence, high nuclei distribution and low nuclei distribution) were identified using supervised receiver operating characteristic (ROC) analysis. The area under the curve (AUC) varies between 0 and 1, where values close to either 0 or 1 indicate discriminatory peptides and values close to 0.5 indicate that the peptides have a similar distribution in the groups. The presence of multiple peptides of the same protein with a similar ROC value (all peptides with AUC ≥ 0.6 ≤ 0.4) was considered a good indicator of the presence of protein. The peptides with an AUC ≥ 0.6 ≤ 0.4 were selected as candidate markers for principal component analysis (PCA) and segmentation using bisecting k-means clustering analysis on the respective groups.

A two-way ANOVA was performed to compare the percentage area occupied by chaotic regions against organised regions and similarly to compare the percentage area occupied by the high against low nuclei distribution regions in the LSCC v/s RSCC. Statistical analysis was performed with Graph Pad Prism 9. A *p*-value of 0.05 was considered as a margin for statistical significance. Data is presented as mean ± SD, * indicates *p* ≤ 0.05, ** indicates *p* ≤ 0.01, *** indicates *p* ≤ 0.001, **** indicates *p* ≤ 0.0001.

Figures were created using the SCiLS Lab software (Bruker, Bremen, Germany, 2024a Pro), biorender, and Inkscape, and graphs were created using GraphPad Prism 9.

## Results

### Imaging protocol for correlation of 2PLSM, MALDI MSI and histology

**Fig. 1:**
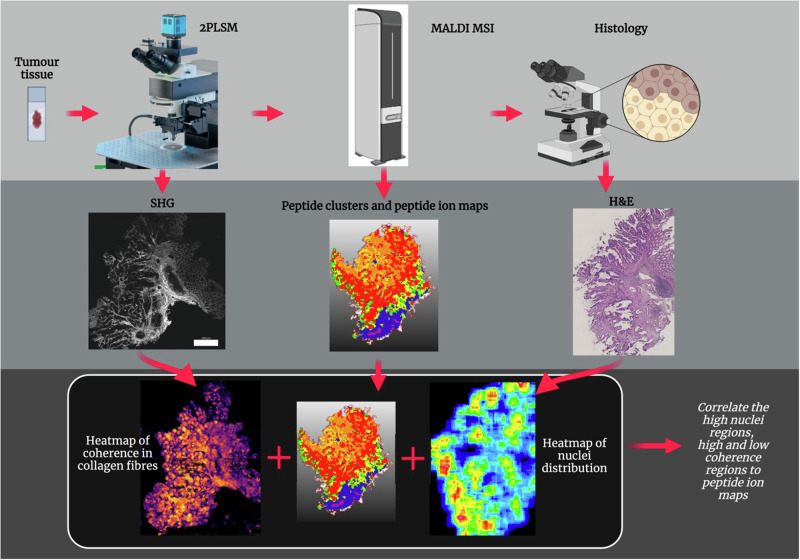
Imaging protocol for correlation of 2PLSM, MALDI MSI and histology. The tumour tissues were first scanned by label-free 2PLSM. The SHG signal generated by the collagen was processed to generate a heatmap depicting the degree of coherence in the collagen fibres. Following 2PLSM, MALDI MSI was performed on the tumour tissues. Spatial heatmaps were generated, depicting the degree of expression of each peptide in the tissue. After MALDI MSI, H&E staining was performed on the tumour tissues. The nuclei segmented from the H&E image were used to generate a heatmap. The peptide expression in the regions of high nuclei distribution and high and low coherence were analysed.

We developed an imaging and analysis pipeline to integrate the imaging features from 2PLSM and histology with the proteomics signatures from MALDI MSI. Initially, we scanned the tissue sections with 2PLSM to capture the SHG and 2PEF signals. Subsequently, we performed MALDI MSI on these sections, followed by H&E staining (Fig. 1). We used the SHG signal emitted by collagen fibres to form a heatmap. This heatmap visually represents the degree of organisation of collagen fibres within the tissue, distinguishing areas based on the coherence of collagen fibres. The regions inside each tissue were categorised into two groups: regions with high coherence of collagen fibres, called ‘organised regions’, and regions with low coherence in collagen fibres, called ‘chaotic regions. Concurrently, we segmented the nuclei from H&E images and formed a heatmap indicating the distribution of nuclei in the tissue. This allowed us to segregate the tissue regions into two groups based on nuclei distribution — regions with high nuclei distribution and regions with low nuclei distribution. Finally, we compared the proteomics signatures across these four morphologically distinct categories — regions with high and low collagen fibre coherence and regions with high and low nuclei distribution.

**Fig. 2:**
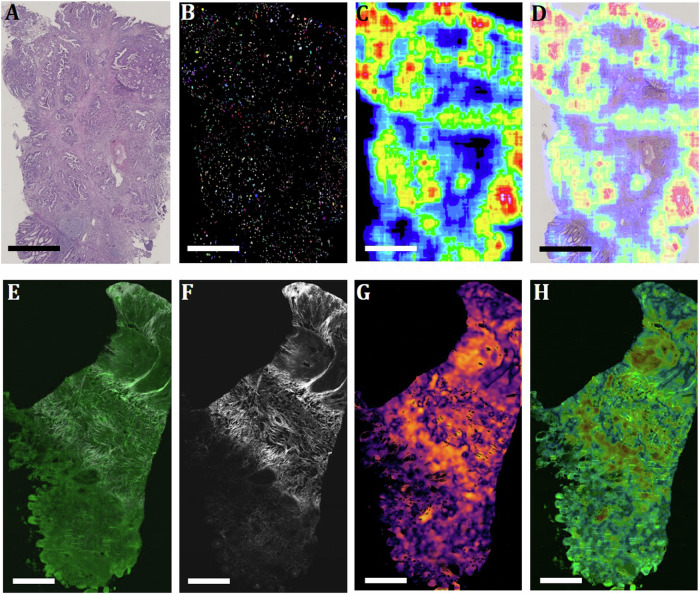
Image analysis pipeline. **A** H&E image of a representative CRC paraffin section depicting tissue morphology. **B** The image shows nuclei segmented from the H&E image. **C** A heatmap is generated from the segmented nuclei and depicts the distribution of nuclei. **D** Overlay of nuclei distribution heatmap and H&E image to annotate the high nuclei distribution regions. **E** 2PLSM image depicting the tissue morphology by the autofluorescence signal (in green) and the collagen-derived SHG signal (in white). **F** Collagen fibre visualisation after extracting the SHG signal. **G** Heatmap depicting the degree of coherence in the collagen fibres generated after texture analysis of the SHG signal. **H** Overlay of a coherence heatmap and a 2PLSM autofluorescence image to annotate the high and low coherence regions. Scale bars in all images represent 1000 µm.

The following image analysis pipeline was introduced to form the heatmaps and annotate the regions (Fig. 2). We first segmented from H&E images (Fig. 2A) the nuclei (Fig. 2B). The number of nuclei per unit area of the tissue was used to form a heatmap, where red represents the regions with the highest nuclei distribution, and blue the regions with the lowest nuclei distribution (Fig. 2C). The heatmap was then overlaid on the H&E image (Fig. 2D).

This merged file was used to annotate the high and low nuclei distribution regions in the original H&E files in QuPath, which were subsequently imported into the SCiLS lab software as classes using the QuPath-SCiLS plugin. Similarly, to form the heatmap of the coherence of collagen fibres, the SHG signal (Fig. 2F) was first extracted from the 2PLSM image (Fig. 2E), where green represents 2PEF or autofluorescence and white represents SHG. The autofluorescence signal emitted from the tissue gives a tissue morphology similar to that of the H&E staining. Texture analysis was performed on the SHG signal, and a heatmap indicating the coherence in collagen fibres was formed (Fig. 2G), where yellow represents regions with high coherence in collagen fibres and violet chaotic regions. The heatmap was then overlaid on the 2PLSM image (Fig. 2H). This merged file was used to annotate the high and low coherence regions in the original H&E files in QuPath, which was subsequently imported into the SCiLS lab software as the classes using the QuPath-SCiLS plugin.

### Discriminative proteomic signatures in LSCC in comparison to RSCC tissue specimens

Univariate analysis of MALDI MSI data resulted in single peptides which are differentially spatially distributed between LSCC and RSCC tumour tissue. 520 aligned *m/z* peaks were determined. From these, 203 *m/z* values could be assigned to 83 proteins from the LC-MS analysis (Supplementary Table 1 and Data 1). Based on these assigned and aligned *m/z* values receiver operator characteristics analysis (ROC) were performed and resulted in discriminative 76 *m/z* values (AUC ≥ 0.6 ≤ 0.4; *p* < 0.01) from 31 corresponding proteins between LSCC and RSCC (Supplementary Table 2). Among the peptides identified as discriminative, several showed particularly high ROC values, indicating an increased spatial intensity distribution in RSCC tumour tissues compared to LSCC.

**Fig. 3:**
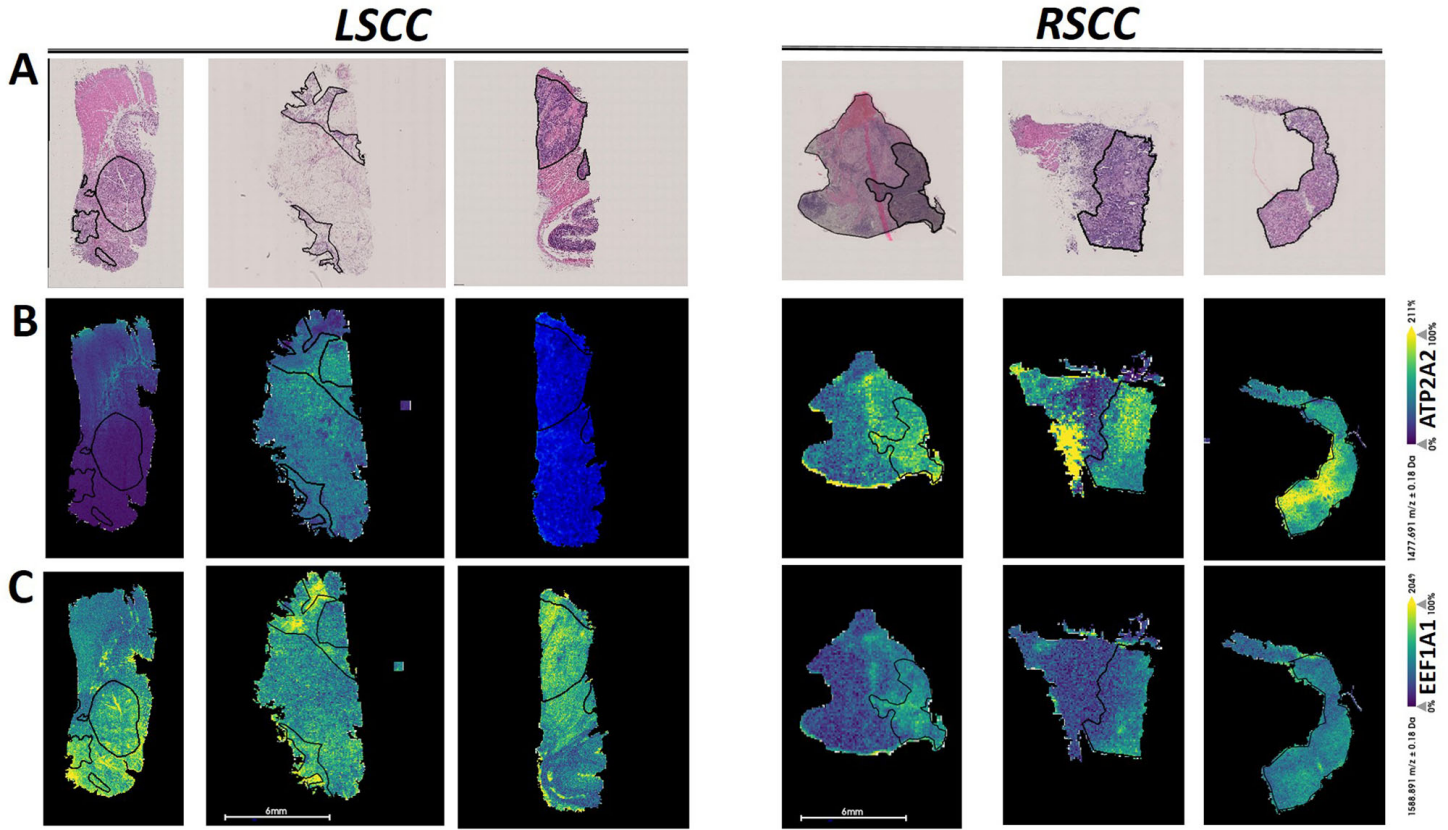
MALDI-MSI discriminative proteomic signatures of LSCC in comparison to RSCC. ROC analysis identified differences in proteomic signatures between LSCC (left-panel) and RSCC (right-panel). **A** Original H&E images of tissues. **B** Increased intensity distribution of *m/z* corresponding to ATP2A2 in RSCC compared to LSCC. **C** Increased *m/z* values corresponding to EEF1A1 in LSCC compared to RSCC shown in the third row. Scale bars represent 6 mm.

Corresponding peptides from ATPase sarcoplasmic/endoplasmic reticulum Ca2+ transporting 2 (ATP2A2, *m/z* 1477, AUC = 0.25), alpha-enolase (ENO1, *m/z* 1962, AUC = 0.26) and vinculin (VLC, *m/z* 1493, AUC = 0.27) showed decreased spatial intensity distribution in LSCC in comparison to RSCC. In contrast, peptides from eukaryotic translation elongation factor 1 alpha 1 (EEF1A1, 1 *m/z* 1588, AUC = 0.66) showed increased spatial intensity distribution (selection is shown in Fig. 3).

Subsequently, functional annotation was performed to annotate the highly represented gene ontology (GO) into the subontology “molecular function” (Supplementary Data 2, and Supplementary Figure 1). The proteins plectin (PLC) and vinculin (VCL) could be determined with a higher intensity distribution in RSCC in comparison to LSCC. These proteins are involved in dystroglycan binding (GO:0002162) which is highly enriched in the dataset. Moreover, the proteins talin-1 (TLN1) and synemin (SYNM) showed a strong interaction in vinculin binding (GO:0017166). Corresponding peptides from vinculin (VLNC) also showed an increased intensity distribution in RSCC in contrast to LSCC.

**Fig. 4:**
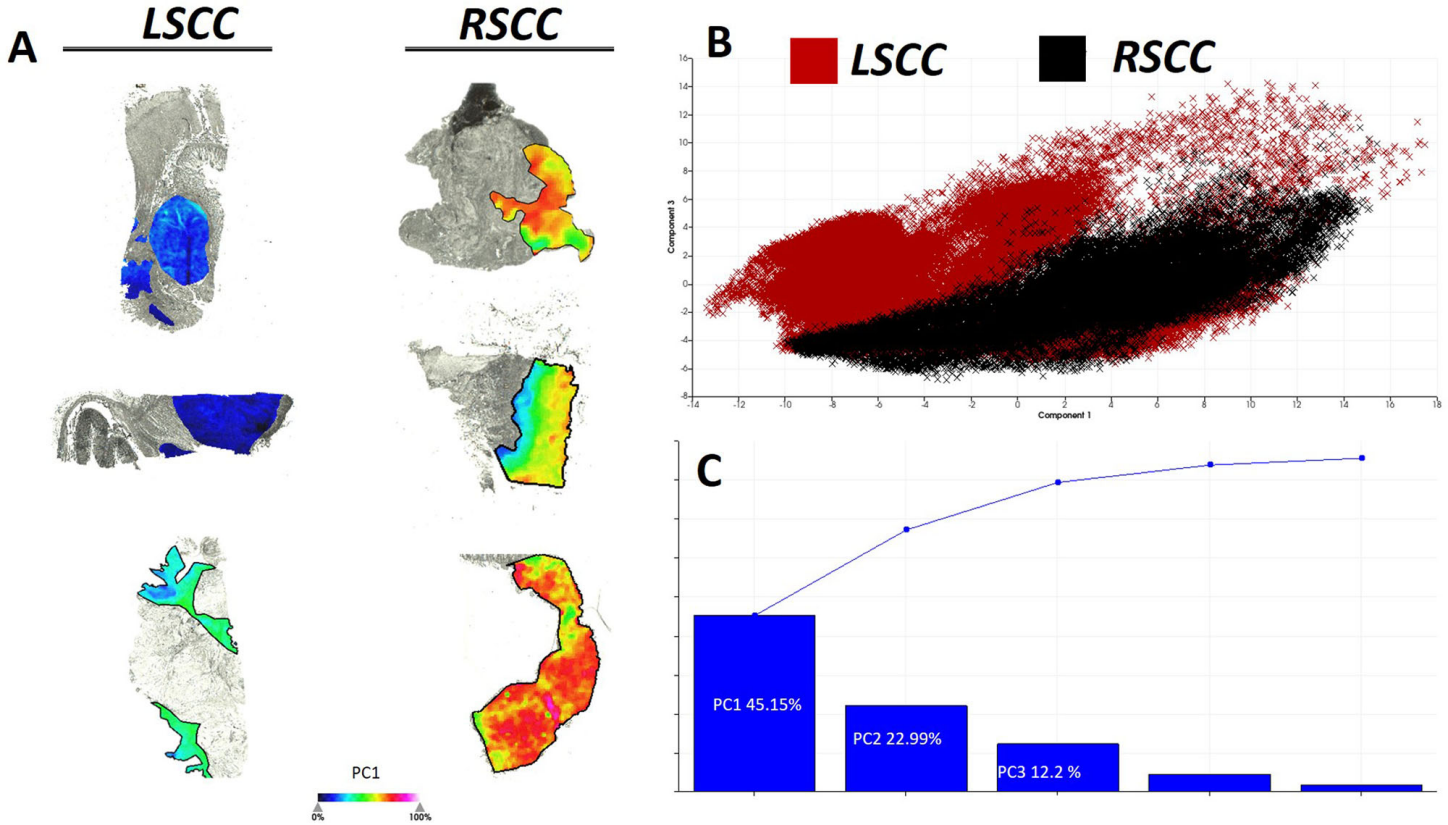
Principle component analysis (PCA) revealed differences in proteomic landscape between LSCC and RSCC. **A** PCA reveals a higher intensity distribution of PC1 in RSCC (right-panel) than that of LSCC (left-panel). **B** The data points in LSCC, shown in red, have a different data point cloud compared to RSCC, shown in black. **C** First three principle components explain 80% of the variability in the two groups.

**Fig. 5:**
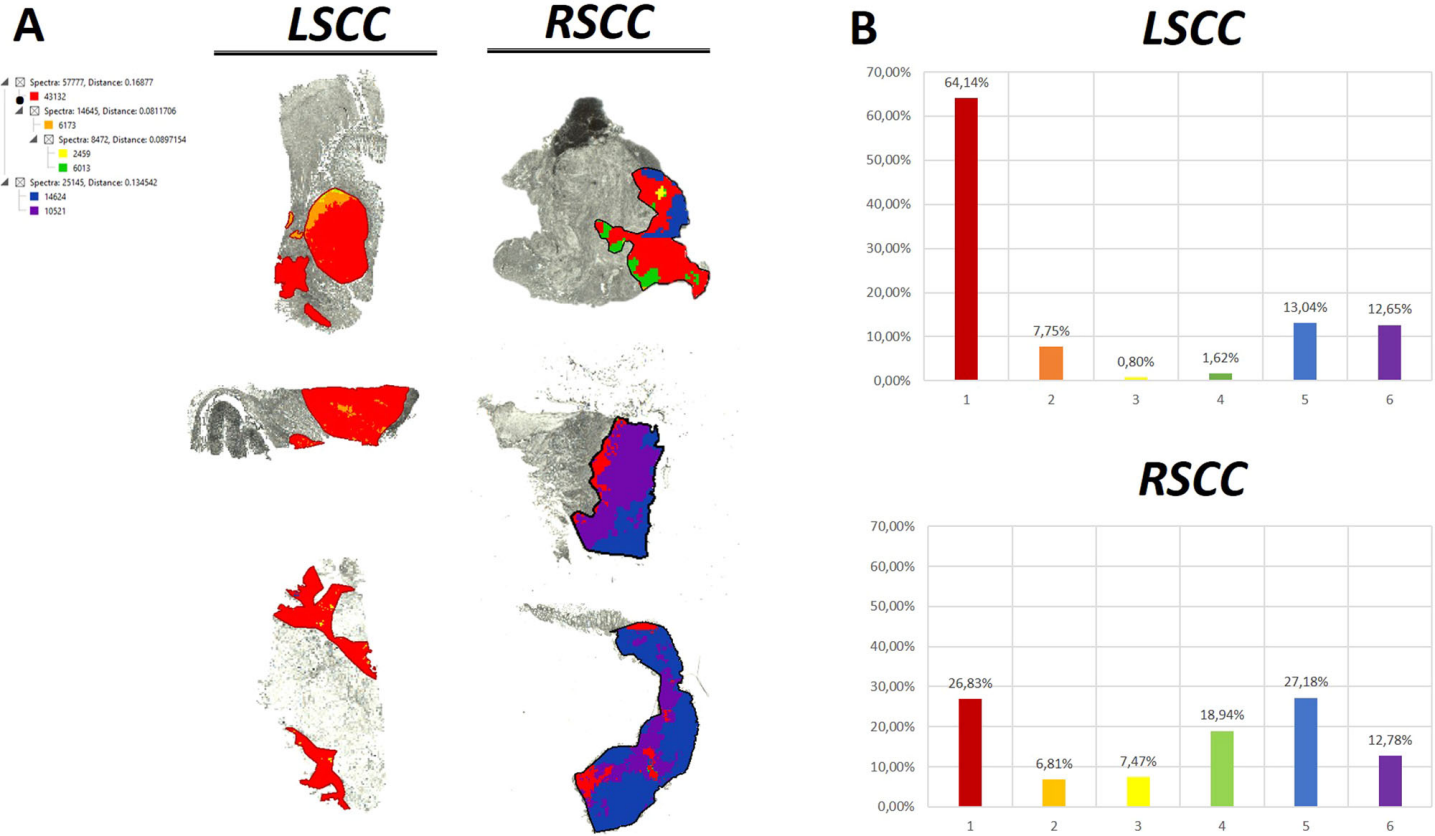
Differences in proteomic clusters between LSCC and RSCC. **A** The proteomic clusters generated by bisecting k-means differ between LSCC (left-panel) and RSCC (right-panel). The spectra and distance of the peptide clusters are shown on extreme left. **B** Quantification of proteomic clusters showed a difference between percentage area occupied by cluster 1 in LSCC and RSCC.

In order to explore the potential of these 76 discriminative *m/z* values, principal component analysis (PCA) was performed (AUC > 0.6 or <0.4). This resulted in an increased intensity distribution of PC1 in the tumour regions of RSCC in comparison to LSCC (selection is shown in Fig. 4A). The loadings plot shows differences between LSCC (Fig. 4B, shown in red) and RSCC (Fig. 4B, shown in black) in the plot of PC1 (x-axis) against PC3 (y-axis). The first three principal components accounted for 80.34% of the variability in the data (Fig. 4C). Subsequently, the discriminative peptides were used to generate proteomic clusters by the bisecting k-means method. The distribution of the generated proteomic clusters (segments indicated by respective colours) showed marked differences between LSCC and RSCC (Fig. 5). The red segment is highly represented in LSCC (64%) in contrast to RSCC tissue (27%). Moreover, the RSCC tumour region includes a higher number of further segments (e.g. blue 27.18%) (Supplementary Table 7).

### High nuclei distribution regions have differentially expressed peptide profiles in LSCC compared to RSCC

**Fig. 6:**
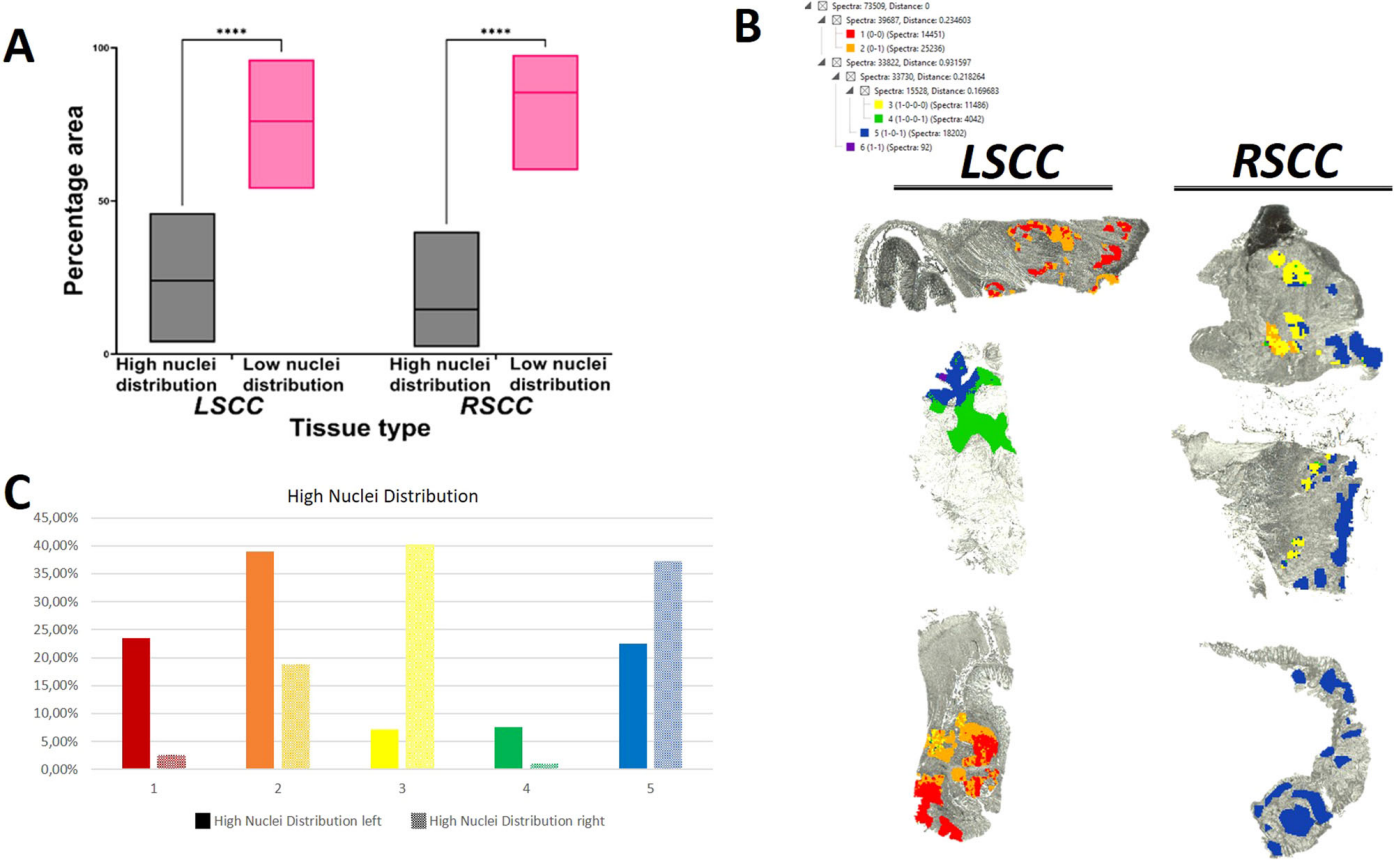
MALDI-MSI identified differences in proteomic signatures in the high nuclei density (HND) regions of LSCC against RSCC. **A** The percentage area occupied by the HND and LND regions in the LSCC and RSCC is shown. **B** Representative images of differential proteomic clusters in the HND regions of LSCC and RSCC, segmented by bisecting k-means are presented. The spectra and distance of the peptide clusters are shown above. **C** Quantification of proteomic clusters shown in **B** highlights differences in percentage area occupied by all five clusters between HND regions of LSCC and RSCC. *N* = 14, **** signifies *p* < 0.0001.

Based on 2PLSM, we found that the high nuclei distribution (HND) regions occupied a significantly lower area (*p* < 0.0001) compared to the low nuclei distribution (LND) regions in both LSCC and RSCC tissues (Fig. 6A). However, the percentage area occupied by the high and low nuclei distribution regions did not differ between LSCC and RSCC. In order to determine differences in proteomics composition of tumour cell-rich, HND regions against the LND, a ROC analysis was performed based on the MALDI MSI data. The paired comparison of the HND against the LND regions determined 14 discriminative *m/z* values (Supplementary Table 4) which corresponded to 7 proteins. Corresponding *m/z* from collagen type I alpha 2 (COL1A2), cathepsin (CTSG), elongation factor 1-alpha 1 (EEF1A1), EH domain-containing protein 2 (EHD2), epiplakin 1 (EPPK1), prelamin-A/C (LMNA), and prolargin (PRELP) showed higher intensity distribution in HND in comparison to LND tumour areas.

Moreover, we performed a paired comparison of HND regions in LSCC against those in RSCC. Out of the 240 aligned and assigned *m/z* values, we determined 65 *m/z* values that were discriminative and identified in the HND regions of LSCC compared to RSCC (Supplementary Table 3). 65 discriminative *m/z* values could be assigned to 25 proteins. Three proteins, caldesmon (CALD), heterogeneous nuclear ribonucleoprotein M (HNRNPM) and tropomyosin 1 (TPM1) were unique discriminative between LSCC and RSCC tumour cell-rich HND regions, however could not be identified as discriminative when the whole LSCC and RSCC tumour region were analysed.

In order to determine the discriminative value of these 65 *m/z* values we generated proteomic clusters by the bisecting k-means method. The distribution of the generated proteomic clusters (segments indicated by respective colours) showed marked differences between the high nuclei distribution regions of LSCC and RSCC (selection is shown in Fig. 6). We quantified the percentage area occupied by the high and low nuclei distribution regions (Fig. 6B, C; Supplementary Table 7).

### Chaotic and organised collagen regions have discriminative peptides in LSCC versus RSCC

**Fig. 7:**
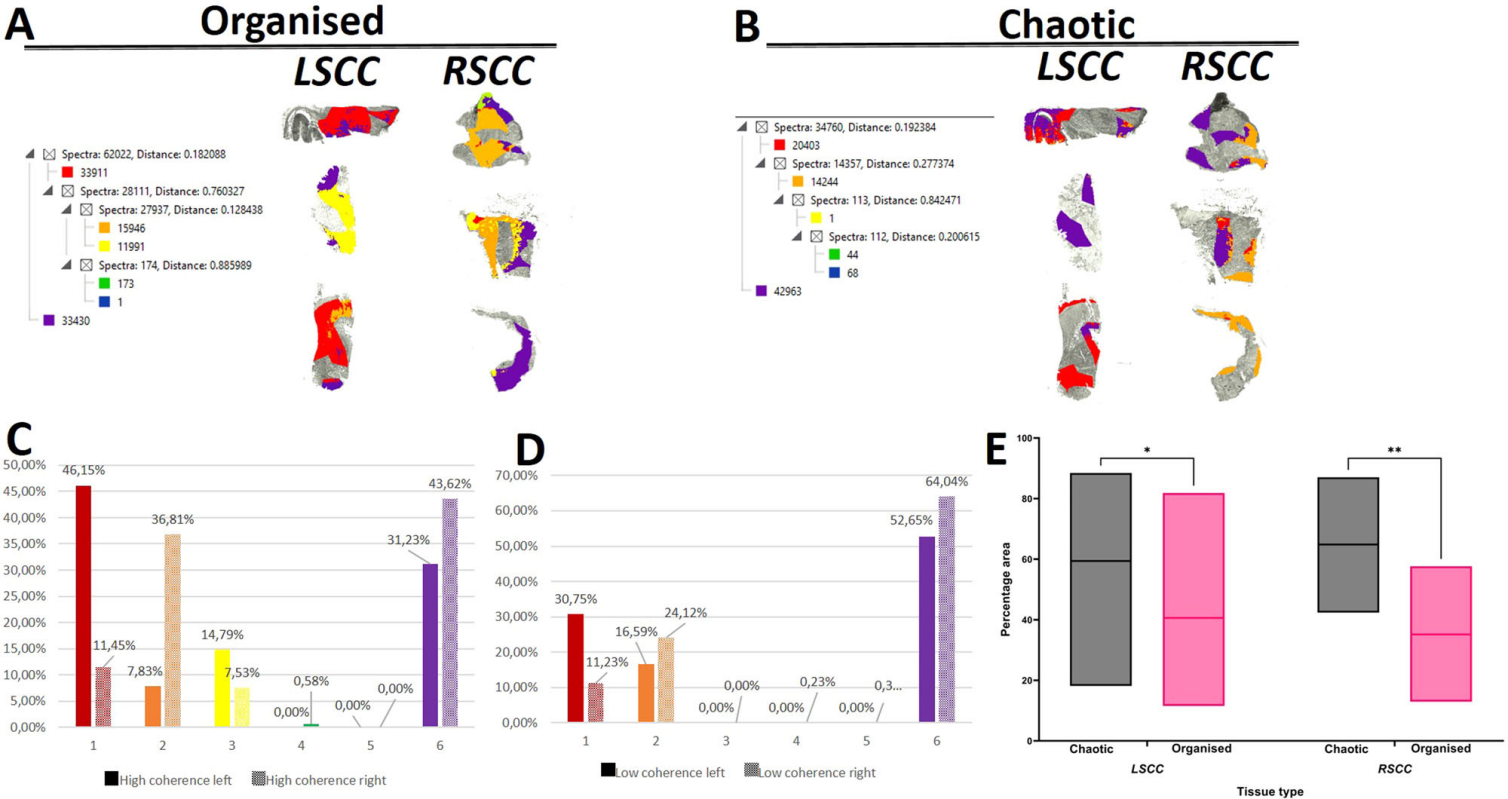
Combination of MALDI-MSI with 2PLSM identifies differential composition of proteomic clusters in chaotic and organised regions of LSCC compared to RSCC. **A** Segmentation by using k-means results in differential proteomic clusters in the organised regions of LSCC compared to RSCC. The spectra and distance of peptide clusters are shown beside the segmentation maps. **B** Segmentation by using k-means results in differential proteomic clusters in the chaotic regions of LSCC compared to RSCC. The spectra and distance of peptide clusters are shown beside the segmentation maps. **C** Quantification of proteomic clusters in the organised regions highlights the differences in percentage areas occupied by each cluster between LSCC and RSCC. **D** Quantification of proteomic clusters in the chaotic regions highlights the differences in percentage areas occupied by each cluster between LSCC and RSCC. **E** The percentage area occupied by the organised and chaotic regions in the LSCC compared to RSCC is shown. *N* = 14, *signifies *p* < 0.05, **signifies *p* < 0.01.

The examination of coherence in the collagen fibre organisation in tumour tissues based on 2PLSM resulted in significant increased percentages of areas with chaotic collagen compared to organised regions within LSCC (*p* = 0.0293) as well as RSCC tumour regions (*p* = 0.0025) (Fig. 7E).

We did not find any differential *m/z* values between high and low collagen coherence regions (data not shown). Using MALDI MSI, the paired comparison of chaotic regions (HC) in LSCC against RSCC by ROC analysis revealed 43 differential *m/z* values (assigned to 19 proteins) (Supplementary Table 5). Among the peptides identified as discriminative were heat shock protein 90 beta family member 1 (HSP90B1, *m/z* 1515, AUC = 0.27), ATPase sarcoplasmic/endoplasmic reticulum Ca2+ transporting 2 (ATP2A2, *m/z* 1477, AUC = 0.27) and non-pou domain containing octamer binding (NONO, *m/z* 1554, AUC = 0.29), indicating a higher intensity distribution of these peptides in RSCC compared to LSCC.

The comparison of organised or low chaotic regions (LC) in LSCC against RSCC by ROC analysis resulted in 63 differential *m/z* values (25 proteins, Supplementary Table 6). Among the peptides identified as discriminative, we found actinin alpha 1 (ACTN1, *m/z* 1961, AUC = 0.19), enolase 1 (ENO1, *m/z* 1962, AUC = 0.21), ATPase sarcoplasmic/endoplasmic reticulum Ca2+ transporting 2 (ATP2A2, *m/z* 1477, AUC = 0.22), heat shock protein 90 beta family member 1 (HSP90B1, *m/z* 1515, AUC = 0.23) indicating a higher intensity distribution of these peptides in RSCC compared to LSCC. In order to investigate the impact of collagen organisation at tumour heterogeneity segmentation was performed based on differential *m/z* values for high and low collagen coherence. Proteomic clusters (segments indicated by respective colours) showed marked differences both in the organised (selection is shown in Fig. 7A) and chaotic regions (selection is shown in Fig. 7B) between LSCC and RSCC. The percentage area occupied by each segment indicated that the proteomic clusters in the organised (Fig. 7C) and chaotic (Fig. 7D) regions of RSCC are composed differently in comparison to LSCC (Supplementary Table 7). For instance, the segment 1 (coloured in red) is much more pronounced in chaotic regions of LSCC in comparison to RSCC.

## Discussion

This study presents a new multimodal imaging approach to characterise the tumour microenvironment (TME) in CRC patient tissues. We show the potential of combining structural information and the nuclei distribution profile with peptide features to identify molecular signatures in different tumour regions to unravel the complex spatial heterogeneity of CRC. MALDI MSI has previously been coupled with various other imaging modalities. Patterson et al. co-registered the autofluorescence signal with MALDI MSI to analyse lipid profiles in murine tissue^27^. In another study combining histology with MALDI MSI, H&E images were trained to differentiate tumour from non-tumour areas and automated annotations were used to compare metabolomic profiles in urachal adenocarcinoma, a rare type of non-urothelial malignancy, with colorectal adenocarcinoma^28^. MALDI MSI signatures were also co-registered with confocal immunofluorescence signals for single-cell analysis by Nikitina et al.^29^. Furthermore, researchers have correlated MALDI MSI with other imaging technologies such as Magnetic Resonance Imaging (MRI)^30^, Fourier Transform Infrared Spectroscopy (FT-IR)^31^ and Raman Spectroscopy^32^. Non-linear image registration has been used before to register adjacent histology sections to MALDI MSI for rapid analysis^33^. Here, we demonstrate, for the first time, a workflow of correlative 2PLSM, histology and MALDI and their potential for obtaining peptide features *(m/z values)* of interest from distinct tumour regions.

Firstly, we investigated the differences in the *m/z* values (peptides) between LSCC and RSCC using univariate testing (ROC analyses). We identified that proteins corresponding to the *m/z* values were associated to molecular functions of proteins, e.g. dystroglycan binding (GO:0002162) and vinculin binding (GO:0017166). Dystroglycan is involved in the cell’s attachment to ECM^34^ and its reduced expression is a predictor of poor outcome in CRC patients^35^. Similarly, loss of cell-cell adhesion due to loss of vinculin and membrane-bound catenin has been shown to promote metastasis and is a predictor of poor prognosis in CRC^36^. Multivariate testing using PCA based on the discriminative identified *m/z* values demonstrate the high discriminative capacity of these peptide signatures. The potential of these *m/z* values in unravelling tissue heterogeneity is shown by using the segmentation. In LSCC, the first component of segmentation is significantly more pronounced than in RSCC.

Spatial heterogeneity in colorectal cancers, e.g. due to the presence of genetically different tumour cells or different immune cell distribution can influence tumour progression and therapy response^37,38^. In order to determine region specific alterations between LSCC and RSCC in the study, we classified the tissues using 2PLSM and histology into four spatially distinct categories: low and high collagen coherence regions as well as low and high nuclei distribution regions. Previous studies have highlighted the importance of analysing the nuclear morphology abnormalities for cancer prognosis. Nuclear morphometry was found to be a prognostic determinant in CRC carcinoma^39–41^. Moreover, benign and CRC tissues have been classified using a random forest algorithm on the features obtained from cell nuclei segmentation on H&E images^42^. Väyrynen et al. identified that a high nuclei distribution of stromal lymphocytes and eosinophils is correlated to a better cancer-specific survival^43^. Researchers have also used nuclear morphometry to categorise high and low-risk breast cancer groups^44^ for the identification of tumour margins^45^ and classification into normal, benign, in-situ carcinoma and invasive carcinoma using convolutional neural networks^46^. These studies advocate for the clinical significance of looking more closely at the high nuclei distribution areas of tumour. In our study, we found *m/z* values corresponding to 6 proteins with a higher intensity distribution in the high nuclei distribution regions compared to low. These are COL1A2, CTSG, EEF1A1, EHD2, EPPK1, LMNA, and PRELP. Some of these, such as collagen, laminin, prolargin, and epiplakin are structural proteins and others such as elongation factor 1 alpha 1 are mainly present in the nucleus. Laminin has been found to promote tumour budding in CRC by interacting with other extracellular matrix (ECM) proteins^47^ and to promote metastasis^48^. EEF1A1 has also been shown to promote CRC progression^49^.

We further analysed the differences in the *m/z* values corresponding to 31 proteins in the high nuclei distribution (HND) areas of LSCC compared to RSCC. The corresponding *m/z* values to Non-POU domain-containing octamer-binding (NONO) protein was found with decreased intensity distribution in the HND of LSCC compared to RSCC. Moreover, exclusive in the HND regions corresponding to *m/z values* from caldesmon 1 (CALD1) and tropomyosin 1 (TPM1) showed higher intensity distribution in RSCC than in LSCC. CALDI1 is a prognostic marker and a potential therapeutic target for stage III/IV pMMR (mismatch repair) CRCs^50^. TPM1 acts as a tumour suppressor and its loss promotes cell proliferation and metastasis by inducing epithelial to mesenchymal transition and regulating cytoskeletal remodelling in CRC^51^.

In a next step we selected “collagen organisation” as the second imaging feature for this study. Multiple studies have underscored the role of collagen fibre organisation in cancer^52^, including our own, which showed significant differences in the collagen fibre amount, waviness and coherence in tumours originating from LSCC against RSCC^14^. Studies have shown that collagen fibres are straighter in cancer tissues^53^, are morphologically distinct in radiotherapy treated v/s untreated patient tissues^54^, and these morphological features of collagen can even be useful in predicting tumour recurrence in CRC patients^55^. These findings underline the need to investigate if proteins are differentially expressed in such spatially distinct regions of collagen fibre organisation. Although no significant differences in the proteomic signatures between chaotic and organised collagen fibre regions were present, by comparing highly and chaotic (low) organised regions of LSCC and RSCC, we found differentially expressed *m/z* values between the two anatomical locations of tumour.

The proteins of corresponding *m/z* values, namely alpha-actinin-1 (ACTN1), Sorbin and SH3 domain-containing protein 1 (SORBS1), talin-1 (TLN1) and plectin (PLEC) are associated with a variety of biological functions such as cell-substrate junction assembly and actin-filament organisation. Vimentin (VIM) was found to be involved in the caspase-mediated cleavage of cytoskeletal proteins, which may directly contribute to the apoptotic changes in cell shape^56^.

The prognosis and response to treatment in CRC patients vary depending on the anatomical origin of the primary tumour, i.e., whether it derived from the left side or the right side of the colon^57^. Since the ECM, in particular collagen organisation, plays a critical role in tumour pathophysiology^58^, analysing the proteomic signatures in highly disorganised in comparison to organised regions of the stroma of CRC tissues might provide insights into the underlying mechanisms by which the disease unfolds. It might also help researchers understand why there is a difference in response to therapy in the two subgroups. In the future, the correlative and multimodal approach introduced in our study could be used to identify novel targets not only in cancer but also in various collagen-associated diseases, such as organ fibrosis of different origins, skeletal and cartilage abnormalities, skin alterations, hearing loss and visual defects, as well as muscle weakness^59^. Our finding that the proteomic landscape in LSCC differs from RSCC tumour regions supports the findings by Hong et al., who found differences in the molecular and immunological hallmarks in the TME of LSCC against RSCC^27^.

However, the comparison of low and high collagen coherence regions in LSCC and RSCC showed that the majority of the discriminative proteins are similar. Only the corresponding peptides from filamin A (FLNA) shows lower intensity distribution exclusively in high collagen coherence in LSCC when compared to RSCC. Filamin A is described as a potential driver of breast cancer metastasis^60^. Moreover, two proteins, actin related protein 3 (ACTR3) and nuclear mitotic apparatus protein 1 (NUMA1) exclusively differ in regions with low collagen coherence showing a lower intensity distribution in LSCC in comparison to RSCC. NUMA1 is related to poor prognosis in esophageal squamous cell carcinoma and ACTR3 is described as associated with colorectal cancer lymph node metastasis^61,62^.

MALDI MSI can be a useful diagnostic tool that may even come forth as a good alternative for multiplexed immunohistochemistry staining, especially if the combination of proteomic signatures is superior in predicting clinically relevant features, such as patient outcome, that can help in guiding the decision of adjuvant chemotherapy. Furthermore, MALDI MSI can be performed in one working day in comparison to other high-throughput techniques, such as a standard molecular analysis with NGS, which needs ten working days to be completed. This highlights its great potential for incorporation into medical settings for a quick, high-throughput spatial molecular analysis.

In conclusion, we developed a new correlative approach by integrating 2PLSM and histology-derived imaging features with MALDI MSI-derived proteomic signatures capable of characterising the TME in CRC patient tissues. By quantifying and comparing the peptide and imaging features in LSCC, RSCC and healthy tissues, we highlight the spatial heterogeneity of the TME and distinguish peptide features in spatially distinct regions of LSCC and RSCC. This study contributes to a better understanding of the TME and introduces a new strategy for identifying proteomic signatures in areas of interest characterised by distinctive imaging features that might correlate to CRC progression, metastasis and treatment response.

## Supplementary information

Supplementary Information1

Supplementary Information2

Supplementary Information

## Data Availability

Data is provided within the manuscript or supplementary information files.

## Code availability

The nuclei segmentation and texture analysis source codes are available at dedicated Github repositories^25,26^.
