# Supplementary material for: MALDI imaging combined with two-photon microscopy reveals local differences in the heterogeneity of colorectal cancer
Source: Npj Imaging. 2024 Sep 23;2:35. doi: 10.1038/s44303-024-00041-3 (PMC12118716; doi:10.1038/s44303-024-00041-3)
Supplement: Supplementary file 3 — Supplementary Information [file 44303_2024_41_MOESM3_ESM.pdf]

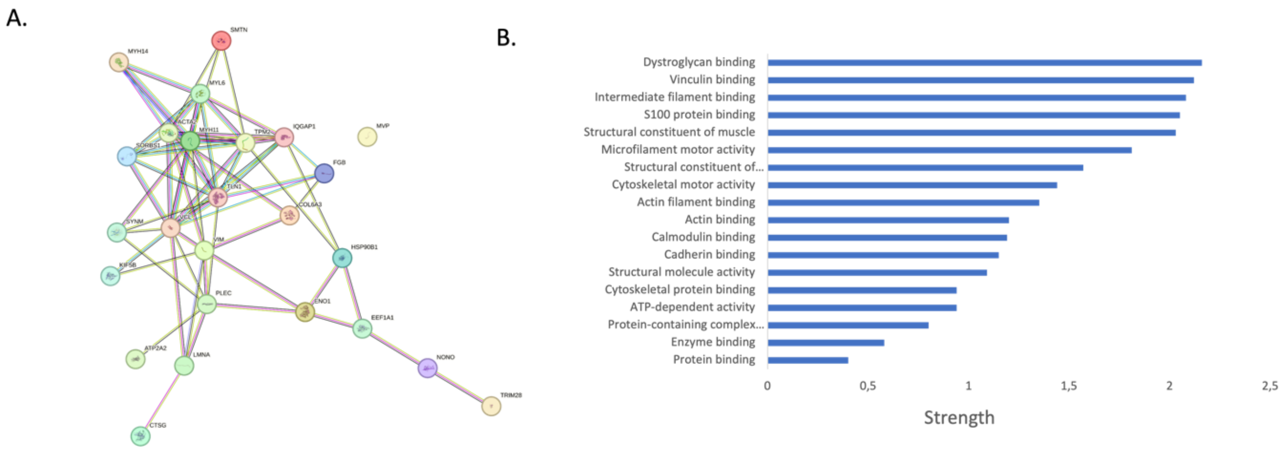

Supplementary Figure 1: STRING analysis of discriminative peptides. A) Protein-protein network of discriminative proteins. B) Functional annotation based on molecular function show the strongest GO terms.

Supplementary Table 1: Aligned MALDI-MSI and LC- MS *m/z* values

MALDI_mass [MH+]	ESI_mass [Mr]	MALDI_mass [Mr]	Distance (ΔDalton)	Gen
700,48	700,39	699,48	0,91	VIM
702,28	701,41	701,28	0,13	IQGAP1
784,48	783,44	783,48	0,04	COL1A2
788,48	787,48	787,48	0	RPL3
792,48	791,42	791,48	0,06	TLN1
795,48	794,47	794,48	0,01	ACTA2
814,48	813,43	813,48	0,04	HSPG2
837,48	836,44	836,48	0,04	KRT18
839,48	838,44	838,48	0,03	EEF1A1
840,48	839,46	839,48	0,02	COL1A2
849,48	848,48	848,48	0	COL6A3
852,48	851,44	851,48	0,04	FGB
859,48	858,48	858,48	0	SMTN
860,08	859,40	859,08	0,33	COL6A2
861,08	860,48	860,08	0,4	TLN1
868,48	867,46	867,48	0,02	COL1A2
869,48	868,50	868,48	0,03	GAPDH
871,48	870,48	870,48	0	EPPK1
879,48	879,43	878,48	0,96	ALB
889,48	888,47	888,48	0,01	CLCA1
895,48	894,46	894,48	0,02	COL1A2
910,48	909,47	909,48	0,01	COL1A2
919,48	918,47	918,48	0	RPS3A
942,48	941,53	941,48	0,05	RPS26
943,48	942,49	942,48	0,02	SYNM
945,48	944,49	944,48	0,02	TPR
965,48	964,48	964,48	0,01	TLN1
966,48	965,48	965,48	0,01	RPL10A
974,48	973,47	973,48	0	LMNA
975,48	974,47	974,48	0	AHNAK
977,48	976,46	976,48	0,01	FLNC
984,48	983,48	983,48	0	ALB
987,48	986,46	986,48	0,01	ACTR3
991,68	990,55	990,68	0,13	TNS1
992,48	991,48	991,48	0	DHX15
994,48	993,51	993,48	0,04	DES
1031,48	1030,49	1030,48	0,01	HSP90B1
1053,48	1052,48	1052,48	0	FLNA
1132,48	1131,47	1131,48	0,01	PLEC
1137,48	1136,51	1136,48	0,04	TRIM28
1139,48	1138,53	1138,48	0,06	EEF1A1
1149,48	1148,56	1148,48	0,08	TPM2

1159,48	1158,57	1158,48	0,1	HNRNPUL2
1163,08	1162,53	1162,08	0,45	PLEC
1184,08	1183,49	1183,08	0,41	CALD1
1193,68	1192,61	1192,68	0,07	MYH9
1199,68	1198,67	1198,68	0,01	TRIM28
1208,68	1207,62	1207,68	0,06	MYH11
1209,68	1208,64	1208,68	0,04	CORO1A
1211,68	1210,67	1210,68	0,01	CLCA1
1214,68	1213,68	1213,68	0,01	COL6A2
1221,68	1220,65	1220,68	0,03	COL1A2
1230,68	1229,68	1229,68	0	FLNC
1241,68	1240,69	1240,68	0,01	LMNA
1243,68	1242,66	1242,68	0,01	AHNAK
1269,68	1268,67	1268,68	0	VCL
1272,68	1271,67	1271,68	0	MYH11
1274,68	1273,68	1273,68	0	EHD2
1290,68	1289,70	1289,68	0,02	MUC2
1296,68	1295,66	1295,68	0,01	CLTC
1302,68	1301,68	1301,68	0,01	KHSRP
1313,68	1312,68	1312,68	0,01	SYNM
1319,68	1318,69	1318,68	0,01	GSN
1323,68	1322,68	1322,68	0	TCOF1
1325,68	1324,68	1324,68	0	SMTN
1335,68	1334,69	1334,68	0,01	TUBB4B
1342,68	1341,67	1341,68	0,01	ATP1A1
1343,68	1342,67	1342,68	0	TPM2
1347,68	1346,62	1346,68	0,05	SMC3
1358,68	1357,69	1357,68	0,01	TF
1363,68	1362,69	1362,68	0,02	MYH11
1366,68	1365,63	1365,68	0,05	TPSB2
1376,68	1375,68	1375,68	0	FLNA
1407,68	1406,68	1406,68	0	RPL27
1427,68	1426,71	1426,68	0,03	HNRNPM
1434,68	1433,69	1433,68	0,01	SYNM
1459,68	1458,70	1458,68	0,02	HNRNPM
1477,68	1476,69	1476,68	0,01	ATP2A2
1480,68	1479,71	1479,68	0,03	KIF5B
1486,68	1485,68	1485,68	0,01	MVP
1488,68	1487,73	1487,68	0,06	TPM1
1493,68	1492,70	1492,68	0,03	VCL
1498,68	1497,68	1497,68	0	SMTN
1500,68	1499,67	1499,68	0	MARCKS
1503,68	1502,70	1502,68	0,02	MYH11
1505,88	1504,81	1504,88	0,06	GBP1
1508,68	1507,68	1507,68	0	CFB
1509,68	1508,66	1508,68	0,01	CFB

1510,68	1509,70	1509,68	0,02	VIM
1515,68	1514,70	1514,68	0,03	HSP90B1
1522,68	1521,71	1521,68	0,03	LMO7
1530,68	1529,68	1529,68	0	COL6A2
1531,68	1530,69	1530,68	0,02	TAGLN
1536,68	1535,67	1535,68	0,01	LMNA
1542,88	1541,86	1541,88	0,02	LMO7
1544,88	1543,84	1543,88	0,04	CTSG
1549,88	1548,86	1548,88	0,02	PRELP
1551,68	1550,67	1550,68	0,01	KIF5B
1552,68	1551,66	1551,68	0,01	LMNA
1554,68	1553,66	1553,68	0,02	NONO
1560,88	1559,83	1559,88	0,04	CTSG
1561,88	1560,85	1560,88	0,02	DES
1563,88	1562,89	1562,88	0,01	MAP4
1579,88	1578,84	1578,88	0,03	RPL10A
1582,88	1581,88	1581,88	0	FLNC
1588,88	1587,87	1587,88	0	EEF1A1
1590,88	1589,89	1589,88	0,01	PRELP
1606,68	1605,69	1605,68	0,02	MYH14
1649,88	1648,89	1648,88	0,02	DES
1652,88	1651,86	1651,88	0,01	CBR1
1655,88	1654,82	1654,88	0,06	CBR1
1656,88	1655,87	1655,88	0	CALD1
1671,88	1670,88	1670,88	0,01	TPM1
1685,88	1684,86	1684,88	0,01	TCOF1
1694,88	1693,88	1693,88	0,01	PPA1
1707,88	1706,88	1706,88	0,01	COL6A3
1710,88	1709,88	1709,88	0	TLN1
1715,88	1714,88	1714,88	0	MYH14
1727,88	1726,88	1726,88	0,01	EHD2
1742,88	1741,89	1741,88	0,01	ALB
1744,88	1743,91	1743,88	0,04	TPR
1745,88	1744,88	1744,88	0,01	MAP4
1750,88	1749,87	1749,88	0	FLNA
1756,88	1755,87	1755,88	0	ACTR3
1757,88	1756,90	1756,88	0,03	TF
1764,88	1763,89	1763,88	0,02	MYH11
1773,88	1772,91	1772,88	0,03	NUMA1
1775,88	1774,90	1774,88	0,02	COL6A1
1788,88	1787,87	1787,88	0,01	MYH11
1789,88	1788,85	1788,88	0,03	MYH11
1790,88	1789,88	1789,88	0,01	ACTA2
1796,88	1795,95	1795,88	0,07	COL6A3
1797,88	1796,88	1796,88	0,01	MUC2
1817,88	1816,95	1816,88	0,07	EPPK1

1819,88	1818,88	1818,88	0	CALD1
1832,88	1831,87	1831,88	0,01	AHNAK
1834,88	1833,89	1833,88	0,01	PLEC
1837,88	1836,88	1836,88	0	ATP2A2
1838,88	1837,86	1837,88	0,02	ATP2A2
1850,88	1849,87	1849,88	0,01	COL1A1
1855,88	1854,90	1854,88	0,02	SORBS1
1888,88	1887,95	1887,88	0,07	TPSB2
1923,88	1922,91	1922,88	0,04	GBP1
1931,88	1930,96	1930,88	0,08	COL6A3
1934,08	1933,01	1933,08	0,07	HSPA5
1952,08	1950,93	1951,08	0,15	SPTAN1
1960,88	1959,90	1959,88	0,03	ACTA2
1962,88	1961,89	1961,88	0,01	ENO1
1968,08	1967,13	1967,08	0,06	COL6A2
1976,08	1975,06	1975,08	0,02	TAGLN
1979,08	1978,07	1978,08	0	GSN
1980,08	1979,06	1979,08	0,02	FLNC
2026,08	2025,11	2025,08	0,04	COL6A2
2038,08	2037,80	2037,08	0,72	SMC3
2041,08	2040,05	2040,08	0,02	TNS1
2042,08	2041,08	2041,08	0,01	VCL
2055,08	2054,08	2054,08	0	IQGAP1
2057,08	2056,07	2056,08	0,01	KHSRP
2058,08	2057,08	2057,08	0	VCL
2072,08	2071,03	2071,08	0,05	XRCC5
2079,08	2078,06	2078,08	0,01	GSN
2083,88	2082,09	2082,88	0,78	HSPG2
2087,08	2086,07	2086,08	0,01	TUBB4B
2088,08	2087,08	2087,08	0,01	DES
2103,08	2102,03	2102,08	0,04	PDLIM1
2105,08	2104,08	2104,08	0,01	PLEC
2114,08	2113,10	2113,08	0,03	KIF5B
2126,08	2125,06	2125,08	0,02	VIM
2136,08	2135,09	2135,08	0,02	MVP
2138,08	2137,08	2137,08	0	RPS3A
2165,08	2163,98	2164,08	0,09	HSPA5
2199,08	2198,07	2198,08	0,01	PLS1
2215,08	2214,06	2214,08	0,01	ACTG1
2216,08	2215,07	2215,08	0	MYH14
2218,08	2217,03	2217,08	0,05	SYNM
2228,08	2227,08	2227,08	0	COL6A3
2229,08	2228,10	2228,08	0,02	GAPDH
2231,08	2230,09	2230,08	0,01	MYH11
2238,08	2237,27	2237,08	0,19	FLNA
2239,08	2237,99	2238,08	0,08	SYNM

2302,08	2301,08	2301,07	0	TAGLN
2303,08	2302,05	2302,07	0,03	NONO
2306,08	2305,06	2305,07	0,01	MYH14
2461,08	2460,11	2460,07	0,04	PPA1
2567,28	2566,26	2566,27	0,02	PALLD
2689,28	2687,31	2688,27	0,97	RPL27
2691,28	2690,34	2690,27	0,07	MYL6
2704,28	2703,39	2703,27	0,12	FLNA
2707,28	2706,34	2706,27	0,07	MYL6
2728,28	2727,33	2727,27	0,06	HSP90B1
2743,28	2742,37	2742,27	0,1	ENO1
2744,28	2743,40	2743,27	0,13	ACTG1
2832,28	2831,38	2831,27	0,11	LPP
2833,28	2832,37	2832,27	0,09	LPP
2870,48	2870,37	2869,47	0,89	KRT18
2871,48	2870,48	2870,47	0,01	RPL3
2873,28	2872,28	2872,27	0	GSN
2875,48	2874,41	2874,47	0,07	SPTAN1
2950,48	2949,44	2949,47	0,03	TRIM28
2951,48	2951,41	2950,47	0,94	PALLD
2961,28	2960,41	2960,27	0,13	ENO1
3101,48	3100,47	3100,47	0	PDLIM1
3123,48	3122,44	3122,47	0,03	XRCC5

Supplementary Table 2: Identified discriminative MALDI-IMS between LSCC and RSCC

MALDI_mass [MH+]	ESI_mass [Mr]	MALDI_mass [Mr]	Distance (ΔDalton)	Gen	AUC [LSCC vs RSCC]
1198,68	1197,70	1197,68	0,02	ACTA2	0,37
1790,88	1789,88	1789,88	0,01	ACTA2	0,38
1960,88	1959,90	1959,88	0,03	ACTA2	0,33
1477,68	1476,69	1476,68	0,01	ATP2A2	0,25
1837,88	1836,88	1836,88	0	ATP2A2	0,34
1838,88	1837,86	1837,88	0,02	ATP2A2	0,34
1796,88	1795,95	1795,88	0,07	COL6A3	0,39
1931,88	1930,96	1930,88	0,08	COL6A3	0,36
2228,08	2227,08	2227,08	0	COL6A3	0,38
1544,88	1543,84	1543,88	0,04	CTSG	0,66
1560,88	1559,83	1559,88	0,04	CTSG	0,61
1588,88	1587,87	1587,88	0	EEF1A1	0,66
839,48	838,44	838,48	0,03	EEF1A1	0,60
1962,88	1961,89	1961,88	0,01	ENO1	0,26
2743,28	2742,37	2742,27	0,1	ENO1	0,38
2961,28	2960,41	2960,27	0,13	ENO1	0,34
2127,08	2126,08	2126,08	0	FGB	0,38
852,48	851,44	851,48	0,04	FGB	0,39
1031,48	1030,49	1030,48	0,01	HSP90B1	0,40
1515,68	1514,70	1514,68	0,03	HSP90B1	0,29
2728,28	2727,33	2727,27	0,06	HSP90B1	0,33
2055,08	2054,08	2054,08	0	IQGAP1	0,40
702,28	701,41	701,28	0,13	IQGAP1	0,38
1480,68	1479,71	1479,68	0,03	KIF5B	0,34
1551,68	1550,67	1550,68	0,01	KIF5B	0,40
1148,48	1147,57	1147,48	0,1	LMNA	0,37
1536,68	1535,67	1535,68	0,01	LMNA	0,30
1486,68	1485,68	1485,68	0,01	MVP	0,36
2136,08	2135,09	2135,08	0,02	MVP	0,40
1503,68	1502,70	1502,68	0,02	MYH11	0,39
1772,88	1771,88	1771,88	0,01	MYH11	0,41
1788,88	1787,87	1787,88	0,01	MYH11	0,36
2231,08	2230,09	2230,08	0,01	MYH11	0,37
1606,68	1605,69	1605,68	0,02	MYH14	0,34
2216,08	2215,07	2215,08	0	MYH14	0,40
2306,08	2305,06	2305,07	0,01	MYH14	0,28
2691,28	2690,34	2690,27	0,07	MYL6	0,29
2707,28	2706,34	2706,27	0,07	MYL6	0,33
1554,68	1553,66	1553,68	0,02	NONO	0,31
2303,08	2302,05	2302,07	0,03	NONO	0,36

1054,48	1053,48	1053,48	0,01	PLEC	0,40
1132,48	1131,47	1131,48	0,01	PLEC	0,37
1834,88	1833,89	1833,88	0,01	PLEC	0,33
2105,08	2104,08	2104,08	0,01	PLEC	0,36
1325,68	1324,68	1324,68	0	SMTN	0,39
1498,68	1497,68	1497,68	0	SMTN	0,35
1321,68	1320,66	1320,68	0,02	SORBS1	0,33
1855,88	1854,90	1854,88	0,02	SORBS1	0,36
1434,68	1433,69	1433,68	0,01	SYNM	0,39
2218,08	2217,03	2217,08	0,05	SYNM	0,39
943,48	942,49	942,48	0,02	SYNM	0,40
1710,88	1709,88	1709,88	0	TLN1	0,36
965,48	964,48	964,48	0,01	TLN1	0,40
1149,48	1148,56	1148,48	0,08	TPM2	0,33
1343,68	1342,67	1342,68	0	TPM2	0,36
1137,48	1136,51	1136,48	0,04	TRIM28	0,34
2950,48	2949,44	2949,47	0,03	TRIM28	0,36
1493,68	1492,70	1492,68	0,03	VCL	0,27
2042,08	2041,08	2041,08	0,01	VCL	0,40
1510,68	1509,70	1509,68	0,02	VIM	0,37
2126,08	2125,06	2125,08	0,02	VIM	0,35

Supplementary Table 3: Identified discriminative MALDI-IMS between high nuclei distribution of LSCC and RSCC

MALDI_mass [MH+]	ESI_mass [Mr]	MALDI_mass [Mr]	Distance (ΔDalton)	Gen	AUC [HND-Left vs right]
795,48	794,47	794,48	0,01	ACTA2	0,38
1198,68	1197,70	1197,68	0,02	ACTA2	0,40
1790,88	1789,88	1789,88	0,01	ACTA2	0,37
1960,88	1959,90	1959,88	0,03	ACTA2	0,33
1477,68	1476,69	1476,68	0,01	ATP2A2	0,20
1837,88	1836,88	1836,88	0	ATP2A2	0,26
1656,88	1655,87	1655,88	0	CALD1	0,40
1819,88	1818,88	1818,88	0	CALD1	0,23
1850,88	1849,87	1849,88	0,01	COL1A1	0,29
1775,88	1774,90	1774,88	0,02	COL6A1	0,25
849,48	848,48	848,48	0	COL6A3	0,62
1796,88	1795,95	1795,88	0,07	COL6A3	0,39
1931,88	1930,96	1930,88	0,08	COL6A3	0,37
1962,88	1961,89	1961,88	0,01	ENO1	0,22
2743,28	2742,37	2742,27	0,1	ENO1	0,36
2961,28	2960,41	2960,27	0,13	ENO1	0,36
1750,88	1749,87	1749,88	0	FLNA	0,37
2238,08	2237,27	2237,08	0,19	FLNA	0,38
1427,68	1426,71	1426,68	0,03	HNRNPM	0,39
1459,68	1458,70	1458,68	0,02	HNRNPM	0,37
1515,68	1514,70	1514,68	0,03	HSP90B1	0,23
2728,28	2727,33	2727,27	0,06	HSP90B1	0,32
1536,68	1535,67	1535,68	0,01	LMNA	0,30
1552,68	1551,66	1551,68	0,01	LMNA	0,33
2832,28	2831,38	2831,27	0,11	LPP	0,29
2833,28	2832,37	2832,27	0,09	LPP	0,30
1503,68	1502,70	1502,68	0,02	MYH11	0,40
1764,88	1763,89	1763,88	0,02	MYH11	0,39
1788,88	1787,87	1787,88	0,01	MYH11	0,32
1789,88	1788,85	1788,88	0,03	MYH11	0,34
2056,08	2055,04	2055,08	0,04	MYH11	0,33
1606,68	1605,69	1605,68	0,02	MYH14	0,34
1715,88	1714,88	1714,88	0	MYH14	0,40
2089,08	2087,99	2088,08	0,08	MYH14	0,32
2216,08	2215,07	2215,08	0	MYH14	0,35
2306,08	2305,06	2305,07	0,01	MYH14	0,26
2691,28	2690,34	2690,27	0,07	MYL6	0,27
2707,28	2706,34	2706,27	0,07	MYL6	0,33
1554,68	1553,66	1553,68	0,02	NONO	0,27
2303,08	2302,05	2302,07	0,03	NONO	0,30
1132,48	1131,47	1131,48	0,01	PLEC	0,39

1834,88	1833,89	1833,88	0,01	PLEC	0,25
2105,08	2104,08	2104,08	0,01	PLEC	0,28
1694,88	1693,88	1693,88	0,01	PPA1	0,39
2461,08	2460,11	2460,07	0,04	PPA1	0,26
1325,68	1324,68	1324,68	0	SMTN	0,38
1498,68	1497,68	1497,68	0	SMTN	0,37
1321,68	1320,66	1320,68	0,02	SORBS1	0,25
1855,88	1854,90	1854,88	0,02	SORBS1	0,29
1531,68	1530,69	1530,68	0,02	TAGLN	0,24
2302,08	2301,08	2301,07	0	TAGLN	0,27
792,48	791,42	791,48	0,06	TLN1	0,65
1710,88	1709,88	1709,88	0	TLN1	0,27
1488,68	1487,73	1487,68	0,06	TPM1	0,33
1671,88	1670,88	1670,88	0,01	TPM1	0,34
1149,48	1148,56	1148,48	0,08	TPM2	0,32
1343,68	1342,67	1342,68	0	TPM2	0,34
1137,48	1136,51	1136,48	0,04	TRIM28	0,36
2950,48	2949,44	2949,47	0,03	TRIM28	0,33
1269,68	1268,67	1268,68	0	VCL	0,39
1493,68	1492,70	1492,68	0,03	VCL	0,22
2042,08	2041,08	2041,08	0,01	VCL	0,31
2058,08	2057,08	2057,08	0	VCL	0,39
1510,68	1509,70	1509,68	0,02	VIM	0,33
2126,08	2125,06	2125,08	0,02	VIM	0,28

Supplementary Table 4: Identified discriminative MALDI-IMS between regions with high and low nuclei distribution

MALDI_mass [MH+]	ESI_mass [Mr]	MALDI_mass [Mr]	Distance [DDalton)	Gen	AUC [HND vs LND]
895,48	894,46	894,48	0,02	COL1A2	0,630
910,48	909,47	909,48	0,01	COL1A2	0,608
1544,88	1543,84	1543,88	0,04	CTSG	0,630
1560,88	1559,83	1559,88	0,04	CTSG	0,609
839,48	838,44	838,48	0,03	EEF1A1	0,625
1588,88	1587,87	1587,88	0	EEF1A1	0,610
1274,68	1273,68	1273,68	0	EHD2	0,611
1727,88	1726,88	1726,88	0,01	EHD2	0,604
871,48	870,48	870,48	0	EPPK1	0,620
1817,88	1816,95	1816,88	0,07	EPPK1	0,619
974,48	973,47	973,48	0	LMNA	0,607
1241,68	1240,69	1240,68	0,01	LMNA	0,626
1549,88	1548,86	1548,88	0,02	PRELP	0,616
1590,88	1589,89	1589,88	0,01	PRELP	0,633

Supplementary Table 5: Identified discriminative MALDI-IMS between chaotic collagen regions in LSCC and RSCC

MALDI_mass [MH+]	ESI_mass [Mr]	MALDI_mass [Mr]	Distance [DDalton)	Gen	AUC [HC-Left vs right]
1832,88	1831,87	1831,88	0,01	AHNAK	0,38
975,48	974,47	974,48	0	AHNAK	0,39
1477,68	1476,69	1476,68	0,01	ATP2A2	0,27
1837,88	1836,88	1836,88	0	ATP2A2	0,33
1796,88	1795,95	1795,88	0,07	COL6A3	0,38
1931,88	1930,96	1930,88	0,08	COL6A3	0,35
1053,48	1052,48	1052,48	0	FLNA	0,40
1750,88	1749,87	1749,88	0	FLNA	0,38
1515,68	1514,70	1514,68	0,03	HSP90B1	0,27
2728,28	2727,33	2727,27	0,06	HSP90B1	0,39
2055,08	2054,08	2054,08	0	IQGAP1	0,40
702,28	701,41	701,28	0,13	IQGAP1	0,36
1536,68	1535,67	1535,68	0,01	LMNA	0,30
1552,68	1551,66	1551,68	0,01	LMNA	0,37
974,48	973,47	973,48	0	LMNA	0,39
1363,68	1362,69	1362,68	0,02	MYH11	0,39
1788,88	1787,87	1787,88	0,01	MYH11	0,38
1789,88	1788,85	1788,88	0,03	MYH11	0,39
1606,68	1605,69	1605,68	0,02	MYH14	0,34
1715,88	1714,88	1714,88	0	MYH14	0,38
2306,08	2305,06	2305,07	0,01	MYH14	0,34
1554,68	1553,66	1553,68	0,02	NONO	0,29
2303,08	2302,05	2302,07	0,03	NONO	0,38
1132,48	1131,47	1131,48	0,01	PLEC	0,38
1834,88	1833,89	1833,88	0,01	PLEC	0,35
2105,08	2104,08	2104,08	0,01	PLEC	0,40
1694,88	1693,88	1693,88	0,01	PPA1	0,38
2461,08	2460,11	2460,07	0,04	PPA1	0,33
1325,68	1324,68	1324,68	0	SMTN	0,36
1498,68	1497,68	1497,68	0	SMTN	0,33
1321,68	1320,66	1320,68	0,02	SORBS1	0,37
1855,88	1854,90	1854,88	0,02	SORBS1	0,38
1434,68	1433,69	1433,68	0,01	SYNM	0,38
943,48	942,49	942,48	0,02	SYNM	0,37
1710,88	1709,88	1709,88	0	TLN1	0,35
965,48	964,48	964,48	0,01	TLN1	0,38
1149,48	1148,56	1148,48	0,08	TPM2	0,34
1343,68	1342,67	1342,68	0	TPM2	0,36
1269,68	1268,67	1268,68	0	VCL	0,36
1493,68	1492,70	1492,68	0,03	VCL	0,30
2042,08	2041,08	2041,08	0,01	VCL	0,38

1510,68	1509,70	1509,68	0,02	VIM	0,37
2126,08	2125,06	2125,08	0,02	VIM	0,39

Supplementary Table 6: Identified discriminative MALDI-IMS between low and (organised) chaotic collagen regions in LSCC and RSCC

MALDI_mass [MH+]	ESI_mass [Mr]	MALDI_mass [Mr]	Distance [DDalton)	Gen	AUC [LC-Left vs right]
1198,68	1197,70	1197,68	0,02	ACTA2	0,37
1790,88	1789,88	1789,88	0,01	ACTA2	0,36
1960,88	1959,90	1959,88	0,03	ACTA2	0,33
1751,88	1750,89	1750,88	0,02	ACTN1	0,36
1961,88	1960,91	1960,88	0,03	ACTN1	0,20
1756,88	1755,87	1755,88	0,00	ACTR3	0,38
987,48	986,46	986,48	0,01	ACTR3	0,37
1832,88	1831,87	1831,88	0,01	AHNAK	0,33
975,48	974,47	974,48	0,00	AHNAK	0,39
1477,68	1476,69	1476,68	0,01	ATP2A2	0,22
1837,88	1836,88	1836,88	0,00	ATP2A2	0,30
1553,68	1552,77	1552,68	0,09	COL1A1	0,28
1850,88	1849,87	1849,88	0,01	COL1A1	0,34
1775,88	1774,90	1774,88	0,02	COL6A1	0,31
1796,88	1795,95	1795,88	0,07	COL6A3	0,36
1931,88	1930,96	1930,88	0,08	COL6A3	0,35
1962,88	1961,89	1961,88	0,01	ENO1	0,21
2961,28	2960,41	2960,27	0,13	ENO1	0,38
1427,68	1426,71	1426,68	0,03	HNRNPM	0,33
1459,68	1458,70	1458,68	0,02	HNRNPM	0,37
1031,48	1030,49	1030,48	0,01	HSP90B1	0,40
1515,68	1514,70	1514,68	0,03	HSP90B1	0,23
2728,28	2727,33	2727,27	0,06	HSP90B1	0,37
2055,08	2054,08	2054,08	0,00	IQGAP1	0,38
702,28	701,41	701,28	0,13	IQGAP1	0,34
1480,68	1479,71	1479,68	0,03	KIF5B	0,31
1148,48	1147,57	1147,48	0,10	LMNA	0,36
1536,68	1535,67	1535,68	0,01	LMNA	0,29
1552,68	1551,66	1551,68	0,01	LMNA	0,32
974,48	973,47	973,48	0,00	LMNA	0,38
2832,28	2831,38	2831,27	0,11	LPP	0,31
2833,28	2832,37	2832,27	0,09	LPP	0,33
1363,68	1362,69	1362,68	0,02	MYH11	0,38
1503,68	1502,70	1502,68	0,02	MYH11	0,38
1764,88	1763,89	1763,88	0,02	MYH11	0,39
1772,88	1771,88	1771,88	0,01	MYH11	0,39
1788,88	1787,87	1787,88	0,01	MYH11	0,33
2056,08	2055,04	2055,08	0,04	MYH11	0,38
1606,68	1605,69	1605,68	0,02	MYH14	0,30
1715,88	1714,88	1714,88	0,00	MYH14	0,36
2306,08	2305,06	2305,07	0,01	MYH14	0,32

1554,68	1553,66	1553,68	0,02	NONO	0,28
2303,08	2302,05	2302,07	0,03	NONO	0,34
1132,48	1131,47	1131,48	0,01	PLEC	0,33
1834,88	1833,89	1833,88	0,01	PLEC	0,31
2105,08	2104,08	2104,08	0,01	PLEC	0,37
1694,88	1693,88	1693,88	0,01	PPA1	0,34
2461,08	2460,11	2460,07	0,04	PPA1	0,28
1325,68	1324,68	1324,68	0,00	SMTN	0,34
1498,68	1497,68	1497,68	0,00	SMTN	0,33
1321,68	1320,66	1320,68	0,02	SORBS1	0,32
1855,88	1854,90	1854,88	0,02	SORBS1	0,33
1434,68	1433,69	1433,68	0,01	SYNM	0,39
943,48	942,49	942,48	0,02	SYNM	0,34
1710,88	1709,88	1709,88	0,00	TLN1	0,33
965,48	964,48	964,48	0,01	TLN1	0,38
1149,48	1148,56	1148,48	0,08	TPM2	0,30
1343,68	1342,67	1342,68	0,00	TPM2	0,35
1269,68	1268,67	1268,68	0,00	VCL	0,35
1493,68	1492,70	1492,68	0,03	VCL	0,26
2042,08	2041,08	2041,08	0,01	VCL	0,34
1510,68	1509,70	1509,68	0,02	VIM	0,32
2126,08	2125,06	2125,08	0,02	VIM	0,38

Supplementary Table 7: Segmentation map of the tumor cell-rich region.

Region	Class 1	Class 2	Clas 3	Class 4	Class 5	Class 6
**LSCC**	35900 (64.14%)	4339 (7.75%)	446 (0.80%)	909 (1.62%)	7300 (13.04%)	7078 (12.65%)
**RSCC**	7232 (26.83%)	1834 (6.81%)	2013 (7.47%)	5104 (18.94%)	7324 (27.18%)	3443 (12.78%)

Region	Class 1	Class 2	Clas 3	Class 4	Class 5	Class 6
**Low coherence LSCC**	18390 (30.75%)	9922 (16.59%)	1 (0.00%)	2 (0.00%)	0 (0.00%)	31487 (52.65%)
**Low coherence RSCC**	2013 (11.23%)	4322 (24.12%)	0 (0.00%)	42 (0.23%)	68 (0.38%)	11476 (64.04%)

Region	Class 1	Class 2	Clas 3	Class 4	Class 5	Class 6
**High coherence LSCC**	30563 (46.15%)	5185 (7.83%)	9791 (14.79%)	2 (0.00%)	1 (0.00%)	20678 (31.23%)
**High coherence RSCC**	3348 (11.45%)	10761 (36.81%)	2200 (7.53%)	171 (0.58%)	0 (0.00%)	12752 (43.62%)

Region	Class 1	Class 2	Clas 3	Class 4	Class 5	Class 6
**High Nuclei Distribution LSCC**	11918 (23.50%)	19754 (38.95%)	3657 (7.21%)	3876 (7.64%)	11428 (22.53%)	82 (0.16%)
**High Nuclei Distribution RSCC**	421 (2.60%)	3057 (18.86%)	6518 (40.20%)	166 (1.02%)	6041 (37.26%)	10 (0.06%)

Supplementary Data 1: LC- MS *m/z* values assigned to MALDI-MSI

Supplementary Data 2: Functional enrichment analysis - Molecular Function
